# Integration of *postmortem* amygdala expression profiling, GWAS, and functional cell culture assays: neuroticism-associated synaptic vesicle glycoprotein 2A (*SV2A*) gene is regulated by miR-133a and miR-218

**DOI:** 10.1038/s41398-020-00966-4

**Published:** 2020-08-24

**Authors:** Magdalena Jurkiewicz, Dirk Moser, Antonius Koller, Lei Yu, Emily I. Chen, David A. Bennett, Turhan Canli

**Affiliations:** 1 Genetics Program, Stony Brook University, Stony Brook, NY USA; 2 Medical Scientist Training Program, Stony Brook University, Stony Brook, NY USA; 3 Integrative Neuroscience, Department of Psychology, Stony Brook University, Stony Brook, NY USA; 4 Proteomics Center, Stony Brook University School of Medicine, Stony Brook, NY USA; 5 Rush Alzheimer’s Disease Center, Rush University Medical Center, Chicago, IL USA; 6 Department of Pharmacological Sciences, Stony Brook University, Stony Brook, NY USA; 7 Department of Psychiatry, Stony Brook University, Stony Brook, NY USA; 8 Present Address: Personalized Genomic Medicine/Department of Pathology and Cell Biology, Columbia University Irving Medical Center, New York, NY USA; 9 Present Address: Department of Genetic Psychology, Faculty of Psychology, Ruhr-University Bochum, Bochum, Germany; 10 Present Address: Proteomics Core Facility, MIT Koch Institute, Cambridge, MA USA; 11 Present Address: Thermo Fisher Precision Medicine Science Center, Cambridge, MA USA

**Keywords:** Molecular neuroscience, Medical genetics

## Abstract

Recent genome-wide studies have begun to identify gene variants, expression profiles, and regulators associated with neuroticism, anxiety disorders, and depression. We conducted a set of experimental cell culture studies of gene regulation by micro RNAs (miRNAs), based on genome-wide transcriptome, proteome, and miRNA expression data from twenty *postmortem* samples of lateral amygdala from donors with known neuroticism scores. Using Ingenuity Pathway Analysis and TargetScan, we identified a list of mRNA–protein–miRNA sets whose expression patterns were consistent with miRNA-based translational repression, as a function of trait anxiety. Here, we focused on one gene from that list, which is of particular translational significance in Psychiatry: synaptic vesicle glycoprotein 2A (SV2A) is the binding site of the anticonvulsant drug levetiracetam ((S)-α-Ethyl-2-oxo-1-pyrrolidineacetamide), which has shown promise in anxiety disorder treatments. We confirmed that *SV2A* is associated with neuroticism or anxiety using an original GWAS of a community cohort (*N* = 1,706), and cross-referencing a published GWAS of multiple cohorts (Ns ranging from 340,569 to 390,278). *Postmortem* amygdala expression profiling implicated three putative regulatory miRNAs to target *SV2A*: miR-133a, miR-138, and miR-218. Moving from association to experimental causal testing in cell culture, we used a luciferase assay to demonstrate that miR-133a and miR-218, but not miR-138, significantly decreased relative luciferase activity from the *SV2A* dual-luciferase construct. In human neuroblastoma cells, transfection with miR-133a and miR-218 reduced both endogenous *SV2A* mRNA and protein levels, confirming miRNA targeting of the *SV2A* gene. This study illustrates the utility of combining *postmortem* gene expression data with GWAS to guide experimental cell culture assays examining gene regulatory mechanisms that may contribute to complex human traits. Identifying specific molecular mechanisms of gene regulation may be useful for future clinical applications in anxiety disorders or other forms of psychopathology.

## Introduction

Neuroticism is a heritable personality trait^1–4^ that shares genetic overlap with anxiety and depression^5–10^. Large-scale genome-wide association studies (GWAS) have identified single nucleotide variants (SNPs) associated with neuroticism, anxiety disorders, and depression^10–12^. Genome-wide expression profiling studies of human brain have begun to identify genes that are differentially expressed as a function of major depressive disorder^13^ or that affect brain structures^14^. Recent work has begun to identify epigenetic regulators of gene expression as potentially useful biomarkers and therapeutic agents in depression^15–19^.

One gene regulatory mechanism of interest with potential therapeutic applications involves microRNAs (miRNAs), which are short (20–23 nucleotides in length), single-stranded, endogenous RNAs that interact with mRNA to regulate gene expression through mechanisms that include mRNA transcript degradation and downregulation of translation of mRNA-to-protein through translational repression^20,21^. Each miRNA can regulate hundreds of genes and affects multiple cellular processes relevant to health and disease including psychiatric and neurological disorders^19,22,23^.

Dysregulation of miRNAs in amygdala has been linked to anxiety-related behaviors in rodent studies^24–27^. Human *postmortem* studies reported elevated expression of miR-155p5 in the amygdala of children with autism spectrum disorder^28^, and decreased expression of miRNA miR-137 in carriers of a schizophrenia risk allele in several brain regions including amygdala^29^. Reviewers have noted a need for a deeper mechanistic understanding of miRNA gene regulation and experimental miRNA manipulation to gauge therapeutic and biomarker potential^23^.

Here, we conducted an experimental analysis of miRNA regulation of the synaptic vesicle glycoprotein 2A (*SV2A*) gene by three putative miRNAs (miR-133a, miR-138, miR-218) in cell culture, based on pilot data we had obtained from a study of *postmortem* amygdala from donors with known neuroticism phenotypes that were cross-validated against a community-based exploratory neuroticism GWAS dataset, and against a published large-scale neuroticism GWAS dataset^10^. Each of the cell culture experiments was independently repeated three times for replication purposes (see “Methods” for details).

## Materials and methods

### *Postmortem* brain samples

Twenty brain donors were participants in the Religious Orders Study and Rush Memory and Aging Project (ROSMAP), a cohort study of common chronic conditions of aging that includes annual cognitive performance tests and clinical evaluations, multiple psychological assessments, and organ donation at the time of death, as described elsewhere^30–34^. Participants signed an informed consent and a Uniform Anatomical Gift Act and the study was approved by the institutional review board of Rush University Medical Center. Participants signed a repository consent that allows their data to be repurposed. The ROSMAP proteomics, mRNA, and miR data are available on the adknowledge portal (adknowledgeportal.synapse.org), an NIA-approved repository. All ROSMAP data can be requested at www.radc.rush.edu.

### Assessment of neuroticism and trait anxiety

Self-reported neuroticism, specifically its sub-facet of trait anxiety, was based on the Revised NEO personality inventory, neuroticism facet 1: anxiety^35^. Individuals who scored in the top quartile had anxiety scores > 16 and were considered anxious, whereas those who scored in the bottom quartile had anxiety scores < 9 and were classified as non-anxious. Sample information is presented in Table 1.

**Table 1:** Sample information, *Postmortem* amygdala.

	Anxious	Control	*T*-test/Chi square
Sample size	10	10	
Anxiety Score (sd)	18.1 (1.8)	4.4 (3.5)	*p* < 2e-9
Sex (Male)	3	4	ns
Age at Death in years (sd)	88.2 (6.0)	88.3 (8.5)	ns
PMI in hours (sd)	7.0 (2.6)	7.0 (2.4)	ns
Antidepressant use	4	4	ns
Anticonvulsant use	3	5	ns

Trait anxious participants and non-anxious controls compared for gender, age at death, *postmortem* interval (PMI), antidepressant use, and anticonvulsant use.

*sd* standard deviation, *ns* non-significant *p* value > 0.05 for Student’s *t* test and Pearson Chi Square where applicable.

### Global expression profiling of the proteome

Frozen human lateral amygdala nucleus samples were prepared with mass spectrometry compatible lysis buffer^36^ and quantified for protein yield. We performed shotgun proteomics analysis (MudPIT), as described elsewhere^37–39^. Briefly, the collected MS spectra were matched to a human protein database from UniProt (database released on January 06, 2012). A decoy database containing the reverse sequences of proteins from the UniProt database was appended to the target database to calculate and filter the results at the false discovery rate of 1% using SEQUEST in the Integrated Proteomics Pipeline (IP2, Integrated Proteomics Inc., CA)^40,41^. Over 2,000 high abundance proteins were identified robustly from each human sample by MudPIT analysis. The data were further integrated and normalized by Scaffold^42^ and relative quantification was derived by normalized spectra counts. Differential expression was defined as a fold change >|1.5| between anxious and control individuals and a Student’s *t* test *p* value < 0.05.

### RNA extraction and mRNA/miRNA profiling

Total RNA was extracted from frozen amygdala tissue using the Qiagen miRNeasy Mini Kit with on-column DNAse treatment (Qiagen, Hilden, Germany). RNA quantity and purity were assessed using a Nanodrop Technologies ND-1000 instrument (NanoDrop Technologies, Wilmington, DE).

For mRNA microarray profiling, total RNA (100 ng) from each individual was prepared as described in the manufacturer’s protocol and analyzed using the Affymetrix U133 Plus 2.0 expression array (Affymetrix, Santa Clara, CA) at the Microarray Core Facility of Stony Brook University. Following washing and staining, arrays were scanned on an Affymetrix model 7G scanner. The scans were analyzed using Affymetrix GCOS. Raw image intensity files were loaded into GenePattern software and normalized using the Robust Multi-Array Average (RMA) method with quantile normalization. Differential expression was defined as a fold change >|1.5| between anxious and control individuals and *t* test *p* value < 0.05. Statistical analyses were conducted using IBM SPSS Statistics version 21.0

For miRNA microarray profiling, total RNA (1 µg) from each individual was prepared as described in the manufacturer’s protocol and analyzed using the Affymetrix GeneChip miRNA 1.0 Array (Affymetrix, Santa Clara, CA). Arrays were scanned and feature extraction was conducted using Affymetrix Command Console software. RMA normalization was conducted using the Affymetrix package of Bioconductor. We did not limit our analysis to human miRNA probes due to significant similarity in sequence between species and possibility for cross-hybridization on the microarray. Differential expression was defined as a fold change >|1.5| between anxious and control individuals and a *t* test *p* value < 0.05. Statistical analyses were conducted using IBM SPSS Statistics version 21.0.

### Identification of mRNA–protein–miRNA sets

We used Ingenuity Pathway Analysis (IPA) (Qiagen, Hilden, Germany) to identify miRNA/protein pairs whose expression pattern across mRNA, miRNA, and protein levels of analysis suggested translational repression of mRNA by miRNAs. First, two lists of proteins and miRNAs, respectively, that were significantly differentially expressed as a function of trait anxiety (defined as a fold change >|1.5| and a Student’s *t* test *p* value < 0.05 between anxious and controls) were uploaded into IPA. IPA software then utilized the computational miRNA target prediction tool TargetScan (www.targetscan.org) to assign pairings between proteins and miRNAs, based on predicted or previously experimentally validated targeting relationships between miRNA and target mRNA. Since we focused on translational repression, we only included gene sets where a given protein and target miRNA showed an inverse expression relationship, and the protein-coding mRNA showed no expression change as a function of trait anxiety status.

### GWAS neuroticism data

SNP data were available from the ROSMAP cohort who had completed a range of personality trait questionnaires. Details on GWAS data generation have been described previously^43^. Briefly, DNA for genotyping was collected from blood, lymphocytes or *postmortem* brain tissue. The majority of samples (*N* = 1,709) were genotyped on the Affymetrix GeneChip 6.0 platform and additional samples (*N* = 384) were genotyped on the Illumina OmniQuad Express platform. Imputation was performed by Alzheimer’s Disease Genetics Consortium on Michigan Imputation Server using the Haplotype Reference Consortium (HRC) reference panel (release 1.1). After quality control, the HRC imputed data were available in 2,182 ROSMAP participants. This dataset was used to identify significant *SV2A* SNPs associated with neuroticism and anxiety, based on a 10-item anxiety questionnaire (*N* = 1,706).

PLINK^44^ was used to test the effects of SNPs with minor allele frequency >1%, including imputed SNPs, to perform set-based tests for all SNPs that mapped to SV2A (±20 kilo-bases). The set-based parameters were *r*^2^ = 0.5, *p* value = 0.05, maximum number of SNPs = 5, and max (T) permutations = 10,000. *SV2A* SNPs identified by PLINK were also cross-referenced against published summary statistics^10^ from the meta-analysis and GWAS for neuroticism and worry, with samples ranging from 340,569 to 390,278 individuals.

### SV2A luciferase assay and site-directed mutagenesis

Dual luciferase assays with an *SV2A* 3′ UTR clone (Genecopoeia, MD) were used to test the hypothesis that the *SV2A* 3′UTR is directly targeted by miR-133a, miR-138, and miR-218, as predicted by Targetscan. The full *SV2A* 3′UTR was cloned downstream of the firefly luciferase reporter gene in a dual luciferase (firefly/renilla) vector. Human embryonic kidney (HEK 293, Sigma-Aldrich, St. Louis, MI) cells under passage 10 were plated in 96-well plates at a density of 5 × 10^4^ cells/well in medium containing Eagle’s Minimum Essential Medium (EMEM) (Life Technologies, Carlsbad, CA) + 2 mM glutamine (Life Technologies, Carlsbad, CA) + 1% non-essential amino acids (NEAA) (Life Technologies, Carlsbad, CA) +10% fetal calf serum (FCS) (Lonza, Allendale, NJ) and co-transfected with 100 ng of the *SV2A*-3′UTR vector and either 100 nM miRNA mimic (miR-133a, miR-218, or miR-138) or 100 nM of miRNA negative control mimic using the DharmaFECT Duo Transfection Reagent (Thermo Fisher Scientific, Waltham, MA). As a negative control, we included *SV2A*-3′UTR vector without miRNA co-transfection.

Transfection was accomplished following the Express Transfection protocol (Thermo Fisher Scientific, Waltham, MA) with 24 h incubation. Luciferase activity was measured using the Dual-Luciferase^®^ Reporter Assay System (Promega, Madison, WI) and the FLUOstarOptima microplate reader (BMG Laboratories, Ortenberg, Germany).

Transfection conditions were optimized using siGLO Green Transfection Indicator (Thermo Fisher Scientific, Waltham, MA). Each experiment was repeated independently three times, and each condition was tested in pentuplicate. Outliers were formally excluded according to Jacobs and Dinman^45^. In each pentuplicate measurement set, between 0 and 2 observations were excluded based on the above criteria. Statistical tests were conducted using one-way analysis of variance (ANOVA) with the Tukey post-hoc test.

To test for site-specific miRNA binding, we conducted site-directed mutagenesis using KOD Xtreme polymerase (Clontech Laboratories, Mountain View, CA). A Targetscan predicted binding site for miR-218 on the full length *SV2A* 3′UTR beginning at position 1051 after the stop codon was mutated from “A**GC**ACA” to “A**CG**ACA” as described elsewhere^46^, and a predicted miR-133a binding site beginning at position 38 after the stop codon was mutated from “GGAC**CAA**A” to “GGA**GGG**AA” in a separate construct. Transfection in HEK 293 cells, which do not endogenously express miR-133/218, was performed as described above, co-transfecting 100 ng of the mutant construct along with 100 nM of the respective miRNA mimic. Each experiment was independently repeated three times, and each condition was repeated in pentuplicate. Statistical analysis of the firefly/renilla ratio followed exclusion of outliers as described above, excluding at most two observations per pentuplicate measurement set. Statistical tests were conducted using one-way ANOVA with the Tukey post-hoc test.

### Neuroblastoma cell culture and transfection

To directly measure *SV2A* mRNA and protein levels as a function of miRNA regulation, 100 nM of miR-133a, miR-138, and miR-218 miRIDIAN miRNA mimics and inhibitors, 100 nM of negative control mimics and 100 nM of negative control inhibitor (Thermo Fisher Scientific, Waltham, MA) were transfected into human neuroblastoma SH-SY5Y cells (Sigma-Aldrich, St. Louis, MI) using Lipofectamine 2000 (Life Technologies, Carlsbad, CA) for 24 h in order to assess changes in mRNA levels, and for 48 hours to assess changes in protein levels. SH-SY5Y cells below passage 10 were plated in six-well plates in pentuplicate (for mRNA analysis) and quadruplicate (for protein analysis) at a density of 5 × 10^5^ cells/well in medium containing Ham’s F12:EMEM (1:1) (Life Technologies, Carlsbad, CA) +2 mM glutamine (Life Technologies, Carlsbad, CA) +1% NEAA (Life Technologies, Carlsbad, CA) +15% FCS (Lonza, Allendale, NJ). Cells were reverse-transfected according to the Lipofectamine 2000 protocol (Life Technologies, Carlsbad, CA). Amounts of lipofectamine and miRNA mimics and inhibitors were optimized using the siGLO Green transfection indicator (Thermo Fisher Scientific, Waltham, MA). Each experiment was independently repeated three times and each condition was repeated in pentuplicate for mRNA analysis and in quadruplicate for protein analysis.

### Real-time quantitative polymerase chain reaction (RT-qPCR)

Total RNA from miR-218, miR-133a, and miR-138 mimic and inhibitor transfected SH-SY5Y cells (see above) was collected using the Qiagen miRNeasy kit using on column DNase treatment according to manufacturer instructions (Qiagen, Hilden, Germany). Reverse transcription was accomplished using the QuantiTect Reverse Transcription Kit (Qiagen, Hilden, Germany) with an input of 250 ng of total RNA for each reaction. RT-qPCR was conducted using the QuantiTect SYBR Green kit with Uracil-N-Glycosylase (UNG) (Qiagen, Hilden, Germany) in a Roche Lightcycler 480 with the following cycling conditions: Step 1: UNG 2 min at 50 °C, Step 2: PCR initial activation 15 min at 95 °C, Step 3 (cycling): denaturation 15 s at 94 °C, annealing 30 s at 60 °C, extension 30 s at 72 °C, Step 4: melting curve analysis. RT-qPCR exon spanning primers were designed using Primer3 with an annealing temperature between 59 and 61 °C^47,48^. Primers were further controlled to specifically target regions which are present in all transcript isoforms using UCSC genome browser (http://genome.ucsc.edu). RT-qPCR normalization was conducted by geometric averaging of multiple internal control genes^49^. Eight housekeeping genes were evaluated for stability with the geNorm algorithm (Biogazelle, Gent, Belgium), identifying *GUSB* (glucuronidase, beta) and *B2M* (beta-2-microglobulin) as fitting controls for mRNA expression analysis in SH-SY5Y cells. GeNorm housekeeping gene selection is depicted in Supplementary Fig. 1, and the RT-qPCR Primer Table is found in Supplementary Table S1. Gene-specific amplification efficiencies were found using the specified procedure in GeNorm software (Biogazelle, Gent, Belgium), and RT-qPCR analysis was conducted using these gene-specific amplification efficiencies. Each experiment was repeated independently three times, each condition was repeated in pentuplicate in each experiment, and each RT-qPCR reaction was performed in triplicate. Outliers were formally excluded as described above, with at most one measurement excluded per pentuplicate set. Statistical tests were conducted using one-way ANOVA with the Tukey post-hoc test.

### Quantitative immunoblotting

One hundred nM miR-218, miR-133a, and miR-138 mimics and inhibitors were transfected into SH-SY5Y cells using Lipofectamine 2000 (Life Technologies, Carlsbad, CA) along with relevant controls as described above. Cells were incubated for 48 h in order to assess changes in SV2A protein levels. Protein was collected using the complete Lysis-M kit (Roche, Basel, Switzerland), and concentrations were established using the Bradford Protein Assay (Bio Rad, Hercules, CA). Quantitative immunoblotting was performed using 100 µg of protein from each condition, which was repeated in quadruplicate. A polyclonal rabbit SV2A antibody (CAT SC28955, Santa Cruz Biotechnology, Santa Cruz, CA) was used at a dilution of 1:100, along with a fluorescently tagged goat anti-rabbit secondary antibody (CAT 611-130-122, Rockland Immunochemicals, Gilbertsville, PA) at a dilution of 1:10,000. A monoclonal mouse β-actin antibody (CAT A4700, Sigma-Aldrich, St. Louis, MI) was used at a dilution of 1:200, along with a fluorescently tagged goat anti-mouse secondary antibody that was used at a dilution of 1:10,000 (CAT 610-131-121, Rockland Immunochemicals, Gilbertsville, PA). SV2A levels were normalized to β-actin to ensure equal loading, and mouse brain lysate served as a positive control to ensure the ability of the antibody to bind SV2A protein. Signal intensity was assessed with the Odyssey Infrared Imaging System, and band quantification was performed with Odyssey Infrared Imaging System Software according to manufacturer instructions (Li-Cor Biosciences, Lincoln, NE). Outliers were formally excluded as described above with at most one measurement excluded per quadruplicate set. Results from three independent experiments were included in the analysis of miR-133a and miR-218, and one independent experiment was done to confirm lack of targeting by miR-138. Statistical tests were conducted using one-way ANOVA with the Tukey post-hoc test.

## Results

### Identification of gene-miRNA sets

The integration of genome-wide expression data yielded 16 mRNA–protein–miRNA sets whose expression patterns were consistent with translational repression as a function of trait anxiety (Supplementary Table S2).

**Fig. 1:**
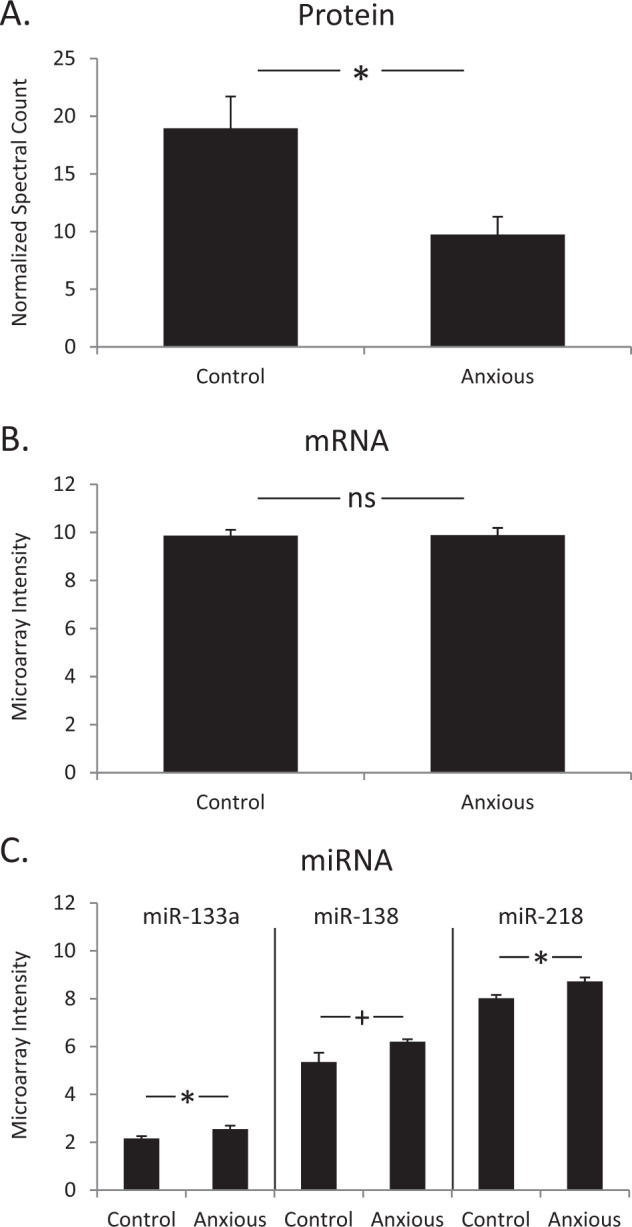
SV2A protein and mRNA levels in human *postmortem* lateral amygdala. **a** Mean SV2A protein levels of anxious individuals compared to controls. Error bars are standard error of the mean (SEM), *t*-test *p* value < 0.05. **b** Mean *SV2A* mRNA levels do not differ significantly between controls and anxious individuals. Error bars are SEM, *t*-test *p* value = 0.87. **c** Significantly higher levels of the three miRNAs (miR-133a, miR-138, and miR-218) are found in anxious individuals, compared to controls. Error bars are SEM, *t-test *p* value < 0.05 for miR-133a and miR-218 expression in anxious individuals versus controls, and + *t*-test *p* value = 0.051 for miR-138 expression in anxious individuals versus controls.

Figure 1 illustrates expression patterns for SV2A protein and mRNA, and three *SV2A*-targeting miRNAs. SV2A protein levels were lower in anxious individuals, compared to non-anxious controls (Fig. 1a) with an observed fold change of 1.94 and *p* < 0.05. However, there was no difference in the transcript level of *SV2A* between these two groups of individuals (Fig. 1b). Although *SV2A* mRNA levels did not differ significantly between the two groups, three *SV2A*-targeting miRNAs (predicted by TargetScan, see Methods), miR-133a, miR-138, and miR-218, were expressed at higher levels in anxious individuals (Fig. 1c).

### *SV2A* in neuroticism GWAS

PLINK identified six SNPs that were significantly associated at nominal levels (*p* < 0.05) with self-reported anxiety in the ROSMAP cohort. Cross-referencing these results against a large-scale published GWAS summary dataset^10^, four of these SNPs were associated with neuroticism and/or worry (Table 2). This summary dataset contained an additional seventy-one *SV2A* SNPs that were significantly associated with neuroticism and/or worry (Supplementary Table S3).

**Table 2:** Cross-referenced SV2A SNPs.

Rush community sample				Nagel (2018): Neuroticism							Nagel (2018): Worry						
Location (Chr:Start)	SNP RSID	*P*	*N*	A1	A2	EAF	MAF	*Z*	*P*	*N*	A1	A2	EAF	MAF	*Z*	*P*	*N*
1:149862367	rs6696191	0.012	1706	A	G	0.11	0.11	1.04	0.298	365,827	A	G	0.10	0.10	−0.33	0.738	341,625
1:149885583	rs577935	0.026	1706	A	G	0.10	0.10	0.19	0.853	388,944	A	G	0.10	0.10	−0.73	0.467	346,980
1:149894445	rs68144650	0.048	1706	A	C	0.92	0.08	3.29	0.001	372,058	C	A	0.08	0.08	−2.86	0.004	347,418
1:149898951	rs16836630	0.049	1706	C	G	0.08	0.08	−3.69	0.000	389,720	C	G	0.08	0.08	−2.92	0.004	347,693
1:149903122	rs72692819	0.049	1706	C	G	0.08	0.08	−3.10	0.002	372,568	C	G	0.08	0.08	−2.73	0.006	347,902
1:149903609	rs12078573	0.049	1706	A	G	0.08	0.08	−3.24	0.001	388,508	A	G	0.08	0.08	−2.24	0.025	346,526
EAF: Effect Allele Frequency									min	365,827						min	341,625
MAF: Minor Allele Frequency									max	389,720						max	347,902

*EAF* effect allele frequency, *MAF* minor allele frequency.

### The SV2A 3′UTR is targeted in a site-specific manner by miR-133a and miR-218, but not miR-138

**Fig. 2:**
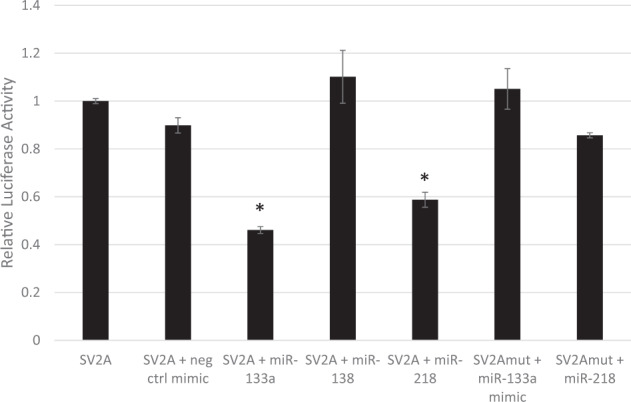
miR-218 and miR-133a but not miR-138 target the 3′UTR of *SV2A*. HEK 293 cells were transfected with the *SV2A*-3′UTR vector (“SV2A”) or *SV2A*-3′UTR mutant vector (“SV2Amut”) and respective miRNA mimics and incubated for 24 h. All firefly/renilla ratios are normalized to the *SV2A* 3′UTR clone. Error bars are SEM, *statistical significance is based on one-way ANOVA with Tukey post-hoc test, *p* value < 0.05 vs SV2A control.

To test targeting of *SV2A* by specific miRNAs identified by genome-wide analysis, we conducted a transfection experiment in HEK 293 cells: transfection with miR-133a and miR-218, but not miR-138, significantly decreased relative luciferase activity from the *SV2A* dual luciferase construct (one-way ANOVA *p* < 0.05) (Fig. 2). Post-hoc Tukey tests showed a significant difference in relative luciferase activity between transfection with the *SV2A* construct only and co-transfection with both the *SV2A* construct and either miR-133a or miR-218 (*p* < 0.00001, for both miRNAs). There was no difference in relative luciferase activity resulting from sole transfection with the *SV2A* construct and co-transfection with the *SV2A* construct and the negative control mimic. To confirm that the predicted target sequences of miR-133a and miR-218 in the *SV2A* 3′UTR are functional, site-directed mutagenesis experiments were performed. Notably, neither miR-133a nor miR-218 could inhibit luciferase activity from the mutagenized *SV2A* construct, suggesting that the predicted sequences are genuine binding sites for the respective miRNAs.

### Transfection with miR-133a and miR-218, but not with miR-138, leads to a reduction in endogenous SV2A mRNA and protein levels

**Fig. 3:**
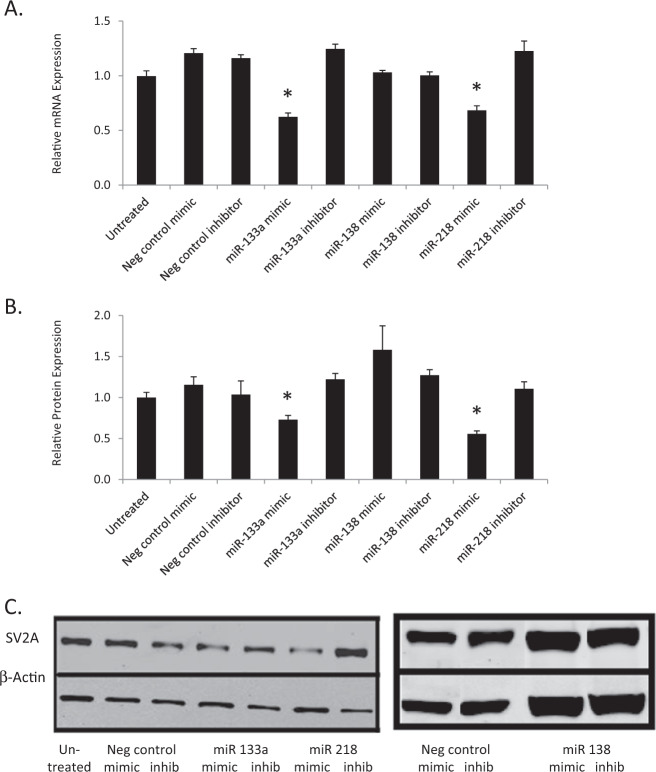
Transfection with miR-133a and miR-218, but not with miR-138, decreases endogenous SV2A mRNA and protein levels in SH-SY5Y cells. **a** Cells transfected with miR-133a and miR-218 mimics, but not miR-138 mimics or other controls, showed a significant decrease in *SV2A* mRNA levels. **b** Cells transfected with miR-133a and miR-218 mimics, but not miR-138 mimics or other controls, showed a significant decrease in SV2A protein levels. **c** The blot images are from representative immunoblots. Error bars are SEM, *statistical significance is based on one-way ANOVA with Tukey post-hoc test, *p*-value < 0.05 vs untreated control condition and mock transfection with negative control mimic.

Given that luciferase assays revealed an interaction between the *SV2A* 3′UTR on the one hand, and miR-133a and miR-218 on the other, we subsequently investigated the effects of miRNA transfection on *SV2A* mRNA and protein levels in human neuroblastoma SH-SY5Y cells. Transfection with either miR-133a or miR-218, but neither with their respective inhibitors nor with miR-138, elicited a significant decrease in *SV2A* mRNA (Fig. 3a) and protein (Fig. 3b, c), compared to controls (one-way ANOVA *p* < 0.05, and Tukey post-hoc *p* < 0.05).

## Discussion

In this study, we leveraged results from a genome-wide expression analysis in *postmortem* human brain tissue with GWAS to identify a target gene associated with neuroticism and anxiety, and to study its regulation through miRNAs in cell culture. Specifically, we utilized *postmortem* amygdala samples from twenty donors with known trait anxiety levels to generate a candidate list of mRNA–protein–miRNA sets whose expression patterns were consistent with translational repression. Of the sixteen sets we found, we focused our cell culture experimental work on the *SV2A* gene, based on its translational potential from prior known associations with anxiety, anxiety disorders, and with epilepsy^50–52^. To address the limitation of small sample size, we confirmed an association for *SV2A* with neuroticism or anxiety in two GWAS sets: one was an original cohort sample of 1,706 individuals, and the other was a published summary dataset^10^ with samples ranging from 340,569 to 390,278 individuals.

There were three differentially expressed miRNAs as a function of trait anxiety (miR-133a, miR-138, and miR-218) predicted to target *SV2A* mRNA. We therefore conducted experimental studies of *SV2A* regulation ex vivo and observed that miR-133a and miR-218, but not miR-138, decreased relative luciferase activity of a *SV2A* dual-luciferase construct in a site-specific manner. Our observation in cell culture that these miRNAs reduced expression of SV2A at both the mRNA and protein level suggests transcript degradation, without excluding translational repression as a possible secondary process. By contrast, the *postmortem* data suggested translational repression only, given that there was no differential expression in mRNA levels. It is possible that the neuroblastoma cell culture system we used is sufficiently different from in vivo conditions that additional processes are engaged to activate transcript degradation in vitro. This is plausible, given that the result of a given miRNA-mRNA interaction can be affected by factors such as binding site accessibility, sequences flanking the miRNA target site and their context, as well as RNA secondary structure, and composition of the miRNA-mediated silencing complex (miRISC), among others^53,54^. Thus, the precise determinants for which mechanism is engaged in the human brain remain to be examined.

*SV2A* is one of three genes of the membrane glycoprotein SV2 and is expressed exclusively in neurons and endocrine cells^55^. SV2A is the most widely expressed isoform^56^ and is the only isoform that is expressed in many GABAergic, inhibitory neurons^56,57^. Rodent studies confirm a role in anxiety. Mice that lack SV2A develop severe seizures and die within 3 weeks of birth^58^, whereas mice heterozygous for one functional copy of *Sv2a* have a normal lifespan, but develop an anxiety-like phenotype^50^. Conditional knockout mice with decreased SV2A in hippocampus are free of epileptic seizures but show elevated levels of anxiety, as measured with the Elevated Plus Maze^59^. Genetically epilepsy-prone rats exhibit an anxiety phenotype across multiple measures prior to having developed epileptic seizures^60^.

Humans have higher levels of SV2A in the amygdala than in other tissues, based on genome-wide expression profiling data^61,62^. SV2A is the binding site of the anticonvulsant drug levetiracetam ((S)-α-Ethyl-2-oxo-1-pyrrolidineacetamide)^52^, which has shown promise in anxiety disorder treatment^63–69^, but also generates states of anxiety as a side-effect in some individuals^70,71^. Such individual differences may reflect underlying genetic variations, which is very plausible, given that multiple *SV2A* SNPs are associated with anxiety by GWAS. Furthermore, SV2A rs626785 produces differentially expressed splice variants in human amygdala (GTEx Analysis Release V8, dbGaP Accession phs000424.v8.p2, sQTL), suggesting additional gene regulatory processes linking anxiety and amygdala gene expression, and possibly epilepsy.

Epilepsy has also been linked to one of the miRNAs studied here. miR-218 (along with miR-204) was significantly down-regulated in hippocampal biopsies from patients diagnosed with mesial temporal lobe epilepsy (MTLE)/hippocampal sclerosis (HS), compared to *postmortem* controls^72^. Experimental manipulation of hippocampal activity was shown to inversely regulate miR-218 expression. Silencing of synaptic activity by tetrodotoxin increased miR-218 expression, whereas induction of sustained synaptic activity with the combination of the GABAA receptor antagonist bicuculline and the K + channel blocker 4-aminopyridine (BiC/4-AP) decreased miR-218 expression^73^. Future studies could be directed to assess levels of miR-133a and miR-218 as risk factors for epilepsy, and in epileptic patients as potential biomarkers for individual differences in response to levetiracetam.

An important limitation of the current work is that the *postmortem* dataset is based on a small sample of donor brains. Thus, the gene expression data remains to be validated in future work with larger samples. The association between SV2A and anxiety, however, is strengthened by leveraging available original and published GWAS data from large-scale samples. Finally, we did not adjust gene expression data for cell type, which could differ between the two *postmortem* anxiety groups.

Future directions could evaluate *postmortem* analysis of a larger dataset of mRNA and proteomic data from the lateral nucleus of the amygdala, as well as other amygdaloid nuclei, as well as other brain regions to determine the specificity of our amygdala results. This work could include histopathological immunolabelling analysis using different markers to identify neuronal sub-populations associated with high trait anxiety. Another line of work could embark on pre-clinical animal studies to examine any causal links between miRs 133a and 218, SV2A, and trait anxiety. Clinical studies of epileptic or anxiety-disordered patients would be useful to address individual differences in responses to the anticonvulsant drug levetiracetam ((S)-α-Ethyl-2-oxo-1-pyrrolidineacetamide)^52^, which is the binding site for SV2A, and for which both beneficial^67–69^ or detrimental^74–80^ effects on emotional states and psychopathology have been reported.

In conclusion, we used a *postmortem* dataset obtained from donors with known trait anxiety phenotypes to generate and identify a set of candidate genes whose expression pattern suggests translational repression by miRNAs. Cross-validation with large-scale GWAS data confirming an association with neuroticism helped us select one gene from this set, *SV2A*, and its associated differentially expressed three miRNAs (miR-133a, miR-138, and miR-218) for further study in cell culture. Although all three miRNAs were computationally predicted to target *SV2A* mRNA, this was experimentally confirmed only for miR-133a and miR-218. This study demonstrates the utility of integrating *postmortem* gene expression profiling with experimental cell culture studies to advance our understanding of gene-regulatory mechanisms related to human brain function and behavior.

## Supplementary information

Supplementary information

Supplementary Table 1

Supplementary Figure 1

Supplementary Table 2

Supplementary Table 3
