# Supplementary material for: Integration of postmortem amygdala expression profiling, GWAS, and functional cell culture assays: neuroticism-associated synaptic vesicle glycoprotein 2A (SV2A) gene is regulated by miR-133a and miR-218
Source: Transl Psychiatry. 2020 Aug 24;10:297. doi: 10.1038/s41398-020-00966-4 (PMC7445165; doi:10.1038/s41398-020-00966-4)
Supplement: Supplementary file 1 — Supplementary information [file 41398_2020_966_MOESM1_ESM.docx]

**Supplementary Information**

**Supplementary Table 1. RT-qPCR primer table.** The primer sequence for each gene is depicted in the table below, along with the amplicon size and sequence.

**Supplementary Table 2. Sixteen sets of protein-mRNA-miRNA triplets.** Table lists fold-change (anxious/controls) for proteins, microRNAs, and mRNAs that are consistent with translational repression as a function of trait anxiety.

**Supplementary Table 3. SV2A GWAS cross-reference.** Table lists *SV2A* SNPs that were nominally significant (*p* < 0.05) in the ROSMAP cohort, and were also significant for neuroticism and/or worry in a published ^1^ GWAS dataset.

**Supplementary Figure 1. *B2M* and *GUSB* are the most stable control genes for RT-qPCR in SH-SY5Y cells as revealed by geNorm analysis.** A). Average expression stability of remaining reference targets. The internal control gene-stability measure M is the average pairwise variation of a particular gene with all other control genes. Genes with the lowest M values are the most stable. Assuming that the control genes are not co-regulated, stepwise exclusion of the gene with the greatest M value results in the combination of two constitutively expressed housekeeping genes that have the most stable expression in tested samples ^2^. *B2M* and *GUSB* show the most stable expression in SH-SY5Y cells. B). Determination of the optimal number of control genes for normalization. Pairwise variation (*Vn*/*n*+1) analysis between the normalization factors NF*n* and NF*n*+1 is done to determine the number of control genes required for accurate normalization. The optimal number of genes for normalization is found below the geNorm Variation (V) threshold of 0.15. Thereby, two genes are sufficient for normalization in this experiment since the addition of a third gene does not contribute to a large variation V. A large variation means that the added gene has a significant effect and should preferably be included for calculation of a reliable normalization factor ^2^.

1. Nagel M, Jansen PR, Stringer S, Watanabe K, de Leeuw CA, Bryois J *et al.* Meta-analysis of genome-wide association studies for neuroticism in 449,484 individuals identifies novel genetic loci and pathways. *Nature genetics* 2018; **50**(7)**:** 920-927.

2. Vandesompele J, De Preter K, Pattyn F, Poppe B, Van Roy N, De Paepe A *et al.* Accurate normalization of real-time quantitative RT-PCR data by geometric averaging of multiple internal control genes. *Genome Biology* 2002; **3**(7).
