# Supplementary material for: Integration of postmortem amygdala expression profiling, GWAS, and functional cell culture assays: neuroticism-associated synaptic vesicle glycoprotein 2A (SV2A) gene is regulated by miR-133a and miR-218
Source: Transl Psychiatry. 2020 Aug 24;10:297. doi: 10.1038/s41398-020-00966-4 (PMC7445165; doi:10.1038/s41398-020-00966-4)
Supplement: Supplementary file 2 — Supplementary Table 1 [file 41398_2020_966_MOESM2_ESM.docx]

| **Gene** | **Sequence 5->3** | **Amplicon size (bp/sequence)** |
| --- | --- | --- |
| Actin, Beta(ACTB)  NM_001101 | Fw: ccaaccgcgagaagatga  Rv: tccatcacgatgccagtg | 121bp  ccaaccgcgagaagatgacccagatcatgtttgagaccttcaacaccccagccatgtacgttgctatccaggctgtgctatccctgtacgcctctggccgtaccactggcatcgtgatgga |
| Beta-glucuronidase  (GUSB)  NM_001284290.1 | Fw: cctgtgacctttgtgagcaa  Rv: aacagatcacatccacatacgg | 70bp  cctgtgacctttgtgagcaactctaactatgcagcagacaagggggctccgtatgtggatgtgatctgtt |
| Beta-2-microglobulin (B2M) NM_004048.2 | Fw: ttctggcctggaggctatc  Rv: tcaggaaatttgactttccattc | 86bp  ttctggcctggaggctatccagcgtactccaaagattcaggtttactcacgtcatccagcagagaatggaaagtcaaatttcctga |
| Glyceraldehyde-3-Phosphate Dehydrogenase (GAPDH) NM_002046.4 | Fw: gctctctgctcctcctgttc  Rv: acgaccaaatccgttgactc | 115bp  gctctctgctcctcctgttcgacagtcagccgcatcttcttttgcgtcgccagccgagccacatcgctcagacaccatggggaaggtgaaggtcggagtcaacggatttggtcgt |
| Hydroxymethylbilane Synthase (HMBS)  NM_000190 | Fw: tgccagagaagagtgtggtg  Rv: agccgggtgttgaggttt | 112bp  tgccagagaagagtgtggtgggaaccagctccctgcgaagagcagcccagctgcagagaaagttcccgcatctggagttcaggagtattcggggaaacctcaacacccggct |
| Succinate Dehydrogenase Complex, Subunit A, Flavoprotein (Fp) SDHA NM_004168.2 | Fw: ggcaacagaagaagcccttt  Rv: caacgtccacataggacagg | 63bp  ggcaacagaagaagccctttgaggagcactggaggaagcacaccctgtcctatgtggacgttg |
| Synaptic vesicle glycoprotein 2A (SV2A) NM_014849.4 | Fw: ccagggttacggcactttc  Rv: gagaagacaatggggatgga | 78bp  ccagggttacggcactttcctcttctgccgcctactttctggggttgggattggagggtccatccccattgtcttctc |
| TATA box binding protein (TBP)  NM_003194.4 | Fw: cggctgtttaacttcgcttc  Rv: cacacgccaagaaacagtga | 75bp  cggctgtttaacttcgcttccgctggcccatagtgatctttgcagtgacccagcatcactgtttcttggcgtgtg |
| Ubiquitin C (UBC)  NM_021009.5 | Fw: ctgatcagcagaggttgatcttt  Rv: gacggagtaccaggtgcaag | 105bp  ctgatcagcagaggttgatctttgccggaaaacagctggaagatggtcgtaccctgtctgactacaacatccagaaagagtccaccttgcacctggtactccgtc |
