# Supplementary material for: Integration of postmortem amygdala expression profiling, GWAS, and functional cell culture assays: neuroticism-associated synaptic vesicle glycoprotein 2A (SV2A) gene is regulated by miR-133a and miR-218
Source: Transl Psychiatry. 2020 Aug 24;10:297. doi: 10.1038/s41398-020-00966-4 (PMC7445165; doi:10.1038/s41398-020-00966-4)

S1 Fig. *B2M* and *GUSB* are the most stable control genes for RT-qPCR in SH-SY5Y cells as revealed by geNorm analysis

A.

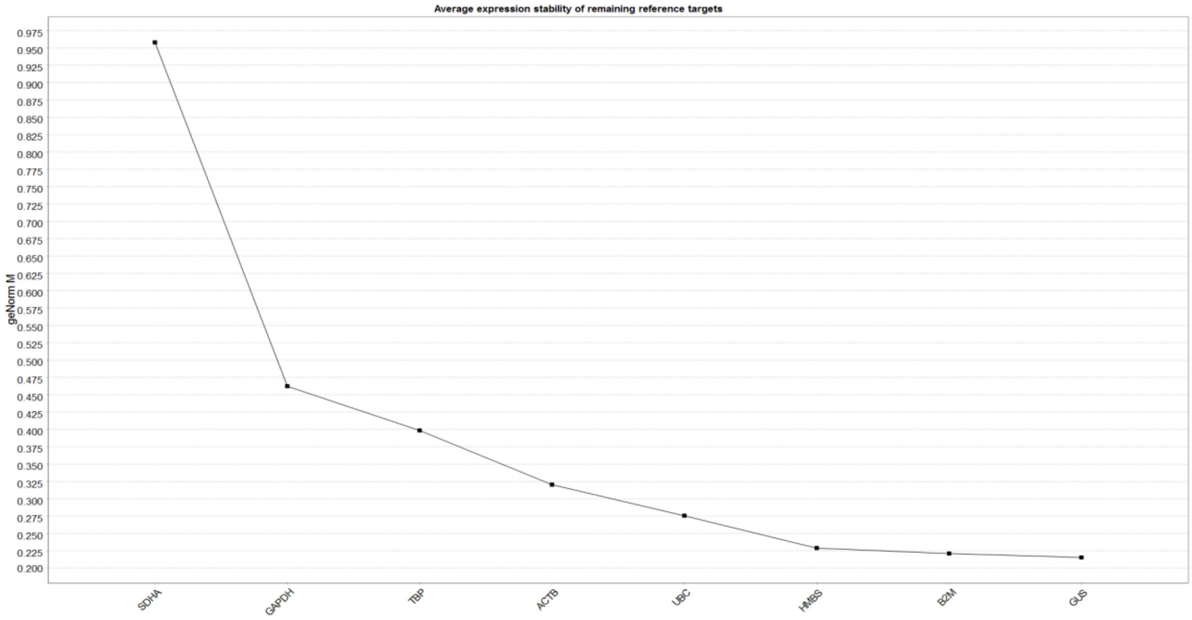

B.

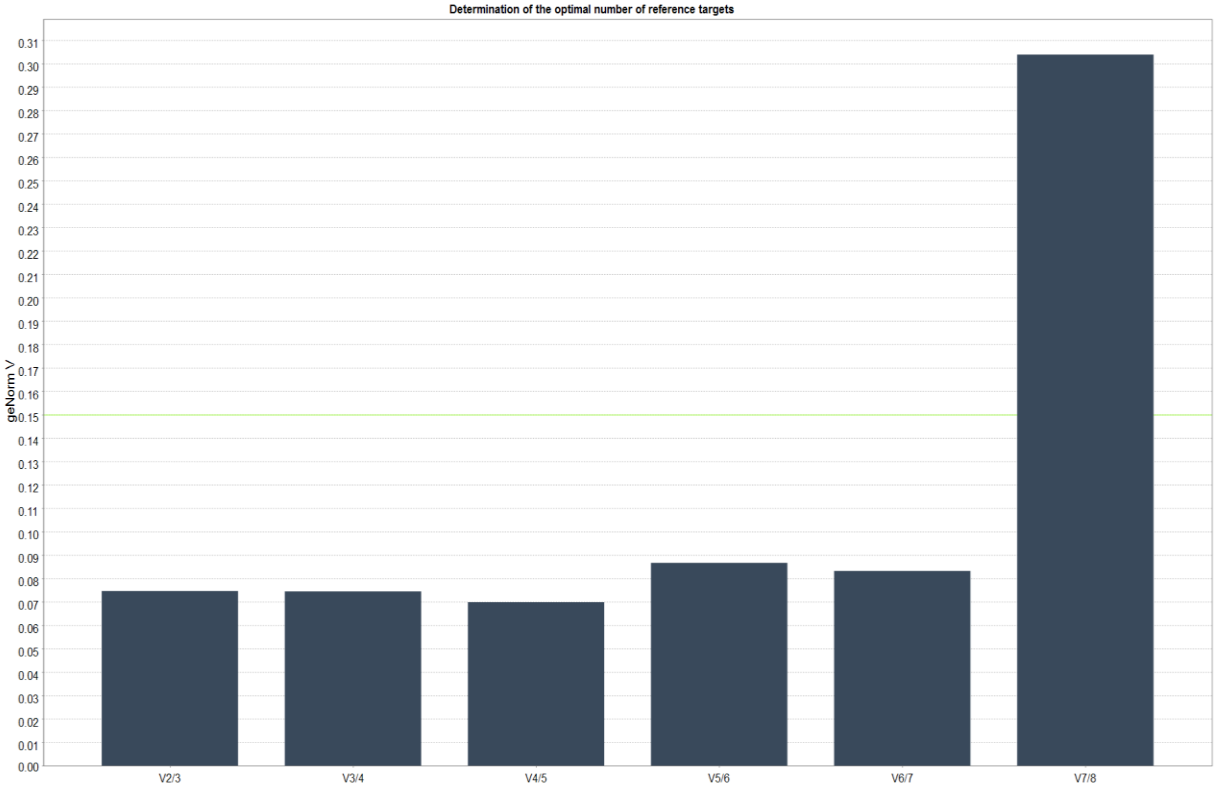

Supplement: Supplementary file 3 — Supplementary Figure 1 [file 41398_2020_966_MOESM3_ESM.pdf]
