# Supplementary material for: Integration of postmortem amygdala expression profiling, GWAS, and functional cell culture assays: neuroticism-associated synaptic vesicle glycoprotein 2A (SV2A) gene is regulated by miR-133a and miR-218
Source: Transl Psychiatry. 2020 Aug 24;10:297. doi: 10.1038/s41398-020-00966-4 (PMC7445165; doi:10.1038/s41398-020-00966-4)
Supplement: Supplementary file 4 — Supplementary Table 2 [file 41398_2020_966_MOESM4_ESM.pdf]

Protein Symbol	Fold Change	miRNA symbol	Fold Change	mRNA symbol	Fold Change
DLGAP3	-3.914	miR-133a-3p	1.534	DLGAP3	1.04
GLS	-3.481	miR-133a-3p	1.534	GLS	-1.22
PPP2R5D	-3.165	miR-133a-3p	1.534	PPP2R5D	1.00
SFXN5	-4.801	miR-133a-3p	1.534	SFXN5	-1.04
		miR-218-5p	1.816		
SV2A	-1.969	miR-133a-3p	1.534	SV2A	1.05
		miR-138-5p	1.522		
		miR-218-5p	1.816		
PHB	-5.480	miR-138-5p	1.522	PHB	-1.02
		miR-205-5p	1.526		
CAMK2A	-2.323	miR-148a-3p	1.561	CAMK2A	-1.05
MPP2	-5.135	miR-148a-3p	1.561	MPP2	-1.09
LETM1	-2.241	miR-205-5p	1.526	LETM1	-1.05
HNRNPA2B1	1.481	miR-217-5p	-1.943	HNRNPA2B1	-1.06
NAP1L1	2.048	miR-217-5p	-1.943	NAP1L1	1.07
SIAE	3.118	miR-217-5p	-1.943	SIAE	1.00
